# Antidepressant mechanisms of ketamine: a review of actions with relevance to treatment-resistance and neuroprogression

**DOI:** 10.3389/fnins.2023.1223145

**Published:** 2023-08-08

**Authors:** August P. M. Lullau, Emily M. W. Haga, Eivind H. Ronold, Gerard E. Dwyer

**Affiliations:** ^1^Department of Biological and Medical Psychology, University of Bergen, Bergen, Norway; ^2^NORMENT Centre of Excellence, Haukeland University Hospital, Bergen, Norway

**Keywords:** major depressive disorder, ketamine, neuroinflammation, neuroprogression, stress, neurotoxicity, neurodegeneration, synaptogenesis

## Abstract

Concurrent with recent insights into the neuroprogressive nature of depression, ketamine shows promise in interfering with several neuroprogressive factors, and has been suggested to reverse neuropathological patterns seen in depression. These insights come at a time of great need for novel approaches, as prevalence is rising and current treatment options remain inadequate for a large number of people. The rapidly growing literature on ketamine’s antidepressant potential has yielded multiple proposed mechanisms of action, many of which have implications for recently elucidated aspects of depressive pathology. This review aims to provide the reader with an understanding of neuroprogressive aspects of depressive pathology and how ketamine is suggested to act on it. Literature was identified through PubMed and Google Scholar, and the reference lists of retrieved articles. When reviewing the evidence of depressive pathology, a picture emerges of four elements interacting with each other to facilitate progressive worsening, namely stress, inflammation, neurotoxicity and neurodegeneration. Ketamine acts on all of these levels of pathology, with rapid and potent reductions of depressive symptoms. Converging evidence suggests that ketamine works to increase stress resilience and reverse stress-induced dysfunction, modulate systemic inflammation and neuroinflammation, attenuate neurotoxic processes and glial dysfunction, and facilitate synaptogenesis rather than neurodegeneration. Still, much remains to be revealed about ketamine’s antidepressant mechanisms of action, and research is lacking on the durability of effect. The findings discussed herein calls for more longitudinal approaches when determining efficacy and its relation to neuroprogressive factors, and could provide relevant considerations for clinical implementation.

## 1. Introduction

Depression remains a global challenge associated with enormous individual and societal costs (James et al., 2018). It is characterized by persistent low mood manifesting as sadness, emptiness, hopelessness or irritation, and anhedonia, or the general loss of ability to feel pleasure and interest. This is often accompanied by weight or appetite changes, insomnia or hypersomnia, psychomotor agitation or retardation, fatigue, feelings of worthlessness or guilt, concentration or decision-making difficulties, and/or suicidal thoughts (American Psychiatric Association, 2022). More than 322 million people are afflicted by this, according to a global report which listed depression as the largest contributor to global disability, and to the yearly suicide prevalence of more than 800,000 deaths (World Health Organization, 2017). Since the release of that report, depression prevalence has been steadily rising each year (Goodwin et al., 2022), increasing by an estimated 27.6% in 2020 as a result of the COVID-19 pandemic (Santomauro et al., 2021).

While serotonergic antidepressants such as the selective serotonin reuptake inhibitor (SSRI) fluoxetine are the most commonly prescribed intervention for depression, the evidence of serotonergic depletion or hypofunction in the disorder was recently considered inadequate (Moncrieff et al., 2022). Importantly, about 50% of depressed patients do not respond to first-line treatment with a conventional antidepressant, and a third still show no response after several trials of different antidepressant medication (Rush et al., 2009). These patients are generally classified as having treatment-resistant depression (TRD), although the conceptualization of TRD varies between different authors, studies, and clinicians (Brown et al., 2019). Rush et al. (2009) defined TRD as having failed at least two treatments of adequate dose and duration. Experts generally do not agree on how to define an adequate dose or duration, however the most frequently cited duration is 4 weeks, and a treatment is generally considered failed if the patient experiences <50% reduction in depressive symptoms (Gaynes et al., 2020). While TRD requirements used in research usually considers failed treatment based on failed pharmacological trials, interviews of clinicians revealed that they universally considered a failed 10–12 week course of psychotherapy as a treatment failure that would contribute to a TRD diagnosis (Brown et al., 2019). The importance of targeting treatment resistance is demonstrated by its association with more time in inpatient care and away from work, more anxiety, stress, sleep disorders, substance use disorders, self-harm, and increased all-time mortality (Lundberg et al., 2023).

Neuroprogressive factors associated with depression could increase the risk of TRD and relapse, with increasing number and duration of episodes presenting a risk in themselves (Moylan et al., 2012). Contributors to neuroprogression include epigenetic alterations, mitochondrial dysfunction, oxidative stress, neurotrophin-, neurotransmitter-, cortisol-, and inflammatory dysfunction, and lifestyle factors such as diet changes (Moylan et al., 2012). The pathophysiological processes associated with depression can ultimately lead to neuronal and glial atrophy (Miguel-Hidalgo and Rajkowska, 2002; Drevets et al., 2008a; MacQueen et al., 2008), which has been associated with the impaired cognition (Maes et al., 2012; Douglas et al., 2018) and dysregulated emotional processing that has been reported in depression (Hamilton and Gotlib, 2008; Grogans et al., 2022; Hu et al., 2022). Furthermore, neurocognitive symptoms, which is associated with greater functional impairment (Jaeger et al., 2006), has been shown to last years into remission (Ronold et al., 2020), with residual cognitive symptoms possibly increasing stress load and vulnerability for relapse (Hammar et al., 2022). As these impairments could increase treatment resistance and challenge attempts to facilitate lasting remission, efforts to identify and treat them are of great importance.

In recent decades, accumulating evidence has revealed ketamine to be a promising tool for rapidly reducing symptoms in treatment-resistant patients (McIntyre et al., 2021). While most commonly described as a non-competitive antagonist of the *N*-methyl-d-aspartate receptor (NMDAR) modulating glutamate, ketamine also targets the opioid, monoamine, and cholinergic systems (McIntyre et al., 2021). Furthermore, research on specific metabolites of ketamine suggests antidepressant effects independently of NMDAR antagonism (Zanos et al., 2016). Ketamine is a racemic compound, consisting of two equal but mirrored molecules called enantiomers, which are broken down into several bioactive metabolites. Multiple lines of evidence have shed light on how effects of standard racemic ketamine could be attributed to its enantiomers or metabolites, an important emerging field beyond the scope of the current review (see Zanos et al., 2016; McMillan and Muthukumaraswamy, 2020; Johnston et al., 2023). Ketamine was first administered to humans in 1964 (Domino et al., 1965), approved by the American Food and Drug Administration for anesthetic use in children, adults, and elderly in 1970, and added to the World Health Organization Model List of Essential Medicines in 1985. Its effects are highly dose-dependent, with doses of 1–2 mg/kg being used in anesthesia, 0.125–0.5 mg/kg in pain management, and 0.5 mg/kg as a starting dose in antidepressant treatment (Kohtala, 2021).

In subanesthetic doses, ketamine has been shown to exert rapid and potent antidepressant effects since the early 2000s (Berman et al., 2000; Lener et al., 2017; Nikkheslat, 2021). Although the evidence is limited by predominantly single-dose designs without long-term follow-up measures, ketamine acutely induces robust reductions of depressive symptoms, especially for treatment resistant individuals (Price et al., 2022). Response rates have been reported to reach as high as 85% at 24 h and 70% at 72 h (Aan Het Rot et al., 2012), with some demonstrating lasting effects 4 weeks following administration (Murrough et al., 2013). In a larger, more recent analysis of ketamine intravenous therapy for depression in real-world settings, 53.6% responded and 28.9% remitted 14–31 days post-infusion (*d =* 1.5), and 73% exhibited reduction of suicidal ideation (McInnes et al., 2022). Ketamine’s efficacy for TRD, its rapid onset of effect, and novel antidepressant mechanisms of action (Lener et al., 2017; McMillan and Muthukumaraswamy, 2020) has garnered excitement within the field of antidepressant research, with some claiming that the drug is revolutionizing the way we think about antidepressant mechanisms (Sanacora and Schatzberg, 2015). Although ketamine’s antidepressant mechanisms are not fully understood, the rapidly growing literature suggests that ketamine acts at multiple levels of pathology, with direct and downstream effects targeting several important points in the depressive self-reinforcing cycle. Importantly, ketamine acts on pathophysiology which could facilitate treatment resistance, such as dysregulated stress-and immune response systems, dysfunctional and atrophied astrocytes, stress-induced atrophy in limbo-cortical networks, and capacity for synaptogenesis.

**Figure 1:**
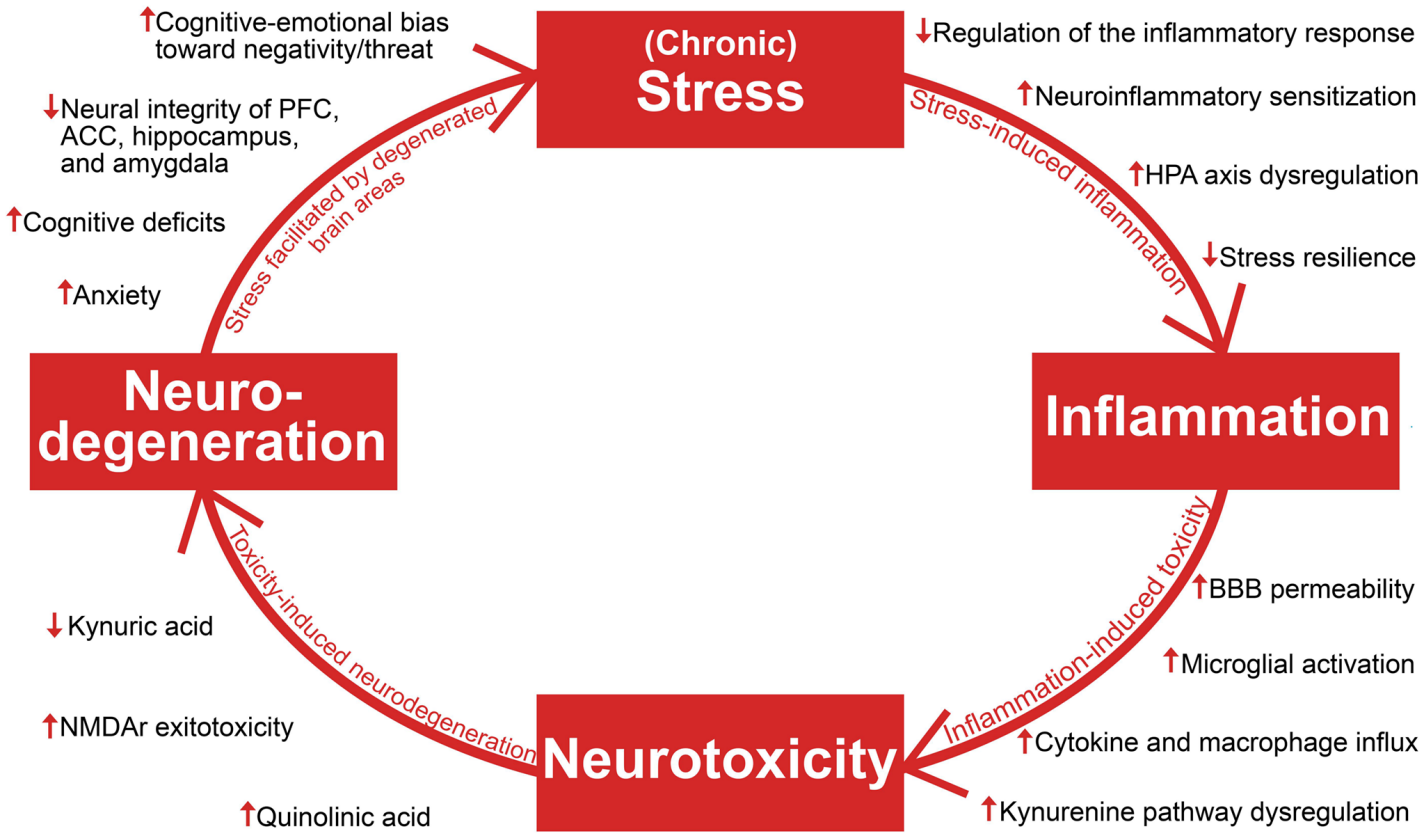
An overview of the reviewed literature on stress, inflammation, neurotoxicity, and neurodegeneration in depression. Viewing depression as a neuroprogressive disorder, the interaction of these four elements and their consequences facilitate progressive worsening of depressive symptomatology. HPA axis, hypothalamic–pituitary–adrenal axis; BBB, blood–brain barrier; NMDAR, *N*-methyl-d-aspartate; PFC, prefrontal cortex; ACC, anterior cingulate cortex.

The aim of this review is to establish an overview of suggested antidepressant mechanisms of ketamine in light of recent insights into the pathophysiology of depression and treatment resistance. The authors opted for a narrative, and not a systematic review based on the goal of synthesizing literature from several different fields of research (with vastly different methodologies) into a clinically meaningful whole, enabling a holistic understanding. Literature searches were performed on PubMed and Google Scholar in the period April 2022 – April 2023, with *Depression, Ketamine, Stress, Inflammation, Neurotoxicity,* and *Neurodegeneration* as main search terms. Additional relevant articles were identified through the reference lists of the included articles, and inclusion of articles was not restricted by their year of publication. Articles whose main focus were bipolar depression or ketamine’s enantiomers or metabolites were not included, as the review focus on patients with major depressive disorder and TRD, and racemic ketamine is and has been the most employed molecule in clinical use and in the relevant body of research. This article provides a description of foundational pathophysiological mechanisms contributing to a progressively worsening depression, brain area and network dysfunction, and degeneration of both glia and neurons (Figure 1). The reviewed literature on ketamine’s antidepressant mechanisms will be discussed in light of the stress and inflammation-related findings which are the focus of the first section, and neurotoxicity and neurodegeneration-related findings which are the focus of the second section.

## 2. Stress and inflammation – a return to homeostasis?

**Figure 2:**
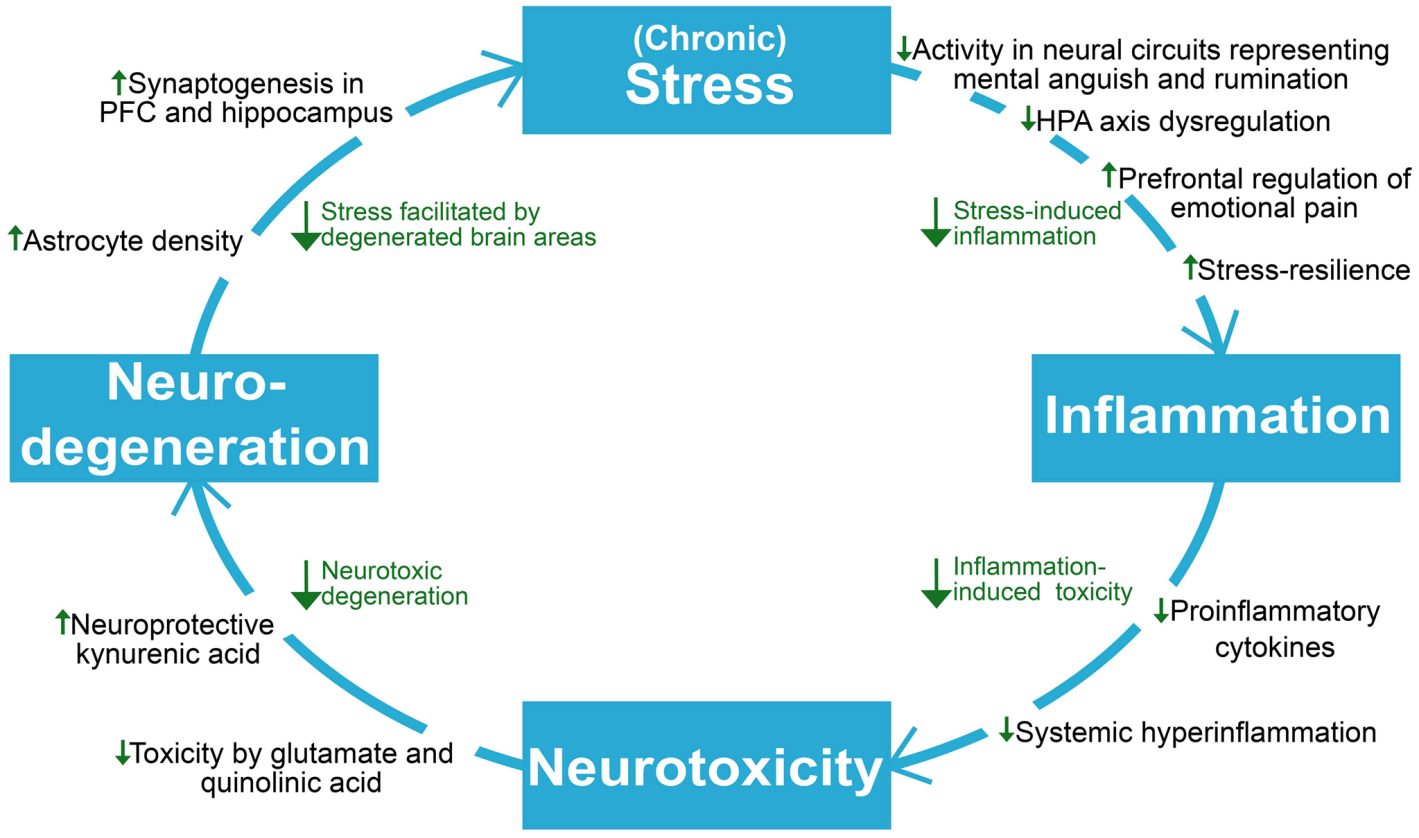
An overview of the reviewed literature on ketamine’s proposed antidepressant mechanisms on neuroprogressive and related factors in depression. Viewing depression as a self-reinforcing cycle of neuroprogression, ketamine seems to act to break several points in this cycle. HPA axis, hypothalamic–pituitary–adrenal axis; PFC, prefrontal cortex.

Depression is causally linked with stress and inflammation, which involves collaborative efforts of several psychobiological systems to respond to threats, damage, and other homeostatic challenges (Hammen, 2005; Osimo et al., 2020). While short-term engagement of the stress and immune systems tend to facilitate adaptation and a return to homeostasis, prolonged activation and failure to terminate their responses can cause severe dysfunction and even death (McEwen, 1998; Barton, 2008; Medzhitov, 2008). As will be explored throughout this section, depression can involve a progressive disruption of the hypothalamic–pituitary–adrenal (HPA) axis, the sympathetic-adrenal-medullary (SAM) axis, the immune system, and the brain areas which are responsible for coordinating biobehavioral adaptation. The continual interplay of these systems makes them interdependent and affected by each other’s pathology, resulting in a complex and bidirectional relationship between stress and inflammation in depression; While psychological stress can cause elevated inflammation and inflammatory pathology (Glaser and Kiecolt-Glaser, 2005; Kim and Won, 2017), inflammation can in turn facilitate increased stress and susceptibility (Vanderzwaag et al., 2023). Importantly, stress-induced alterations of glucocorticoid function and sensitivity can hinder HPA axis-regulation of stress and inflammation and reprogram immune cells towards an increasingly proinflammatory state, which can cause degeneration of brain areas involved in stress-and inflammatory responses (McGowan et al., 2009; Slavich, 2020; Crișan et al., 2016). The SAM axis has also been implicated in stress-induced inflammation through its ability to upregulate cytokine release through noradrenaline and its possible contribution to neuro-inflammatory sensitization following adversity (Irwin and Cole, 2011; Slavich and Irwin, 2014). The elucidation of stress-immune interactions in depression become increasingly relevant for understanding ketamine’s antidepressant mechanisms, which seems to involve interruption of maladaptive dynamics between these systems. Ketamine’s role as a unique homeostatic regulator of inflammation and stress-induced immune dysfunction (De Kock et al., 2013) has wide spanning implications for the highly entangled elements maintaining depression, as interruption of one link in the system might attenuate downstream effects contributing to progressive worsening (Figure 2).

### 2.1. Alterations of the HPA axis and stress resilience

When the limbic and lower brain regions sense a significant threat to homeostasis, they signal to the hypothalamus which then releases corticotropin-releasing hormone (CRH), whose downstream effects cause release of glucocorticoids like cortisol (Smith and Vale, 2006). While the HPA axis is normally self-regulated through negative feedback inhibition by glucocorticoids, chronic stress can lead to glucocorticoid resistance and a failure of this inhibition (Charmandari et al., 2008). The disrupted glucocorticoid signaling causes a gradual shift towards higher homeostatic set points of the axis, resulting in continuous overactivation (Holsboer, 2000). This is demonstrated in depressed individuals, who exhibit increased production and secretion of cortisol, with an increased cortisol response to awakening, and a blunting of the normal dip that should occur in the evening (Pariante and Lightman, 2008; Vreeburg et al., 2010). Furthermore, HPA axis dysregulation is associated with suicide, interacting with factors such as trauma, cognitive impairment, sleep disruptions, and epigenetic influences to increase suicide risk (O’Connor et al., 2020). Likewise, several biopsychosocial and epigenetic factors interact to increase risk of stress and depression. For example, early life stress is associated with epigenetic alterations of the NR3C1 gene encoding the glucocorticoid receptor (GR), with subsequent glucocorticoid resistance and development of depression (Palma-Gudiel et al., 2015). Recently, similar alterations of the gene were also found in infants of cold or hesitant mothers, and persisted until the children were assessed 6 years later (Holdsworth et al., 2023). These results present a possible mechanism explaining previous findings that uncertain childhood environments and adversity can cause sustained dysfunction in CRH-releasing neurons, and increase vulnerability to stressors later in life (Arborelius et al., 1999). While findings demonstrate long-lasting dysfunction, it is important to note that this is not necessarily permanent. Several antidepressants have been shown to directly affect GRs and increase their expression and ability to accomplish negative feedback inhibition, suggesting that reversal of this stress-induced dysfunction is possible (Pariante and Miller, 2001).

Rodent models have suggested ketamine’s effect to involve increased stress resilience, and show direct effects on components of the HPA axis. In rats subjected to chronic social defeat stress, ketamine decreased depressive-like behavior, which was attributed to a normalization of the HPA axis response (Wang et al., 2019). This involved a reduction of plasma corticosterone and stress-induced abnormalities of GR expression and nuclear translocation in the hippocampus. Ketamine has also been demonstrated to have protective effects when administered before stress (Brachman et al., 2016). In this study, mice were administered a large injection of ketamine and subjected to lasting stress or corticosterone administration. Ketamine protected against development of social avoidance, decreased exploration, learned helplessness, latency to feed and groom, and signs of behavioral despair. The team also investigated whether the common SSRI, fluoxetine, was able to exert similar protective effects. Three weeks of fluoxetine treatment showed no effect on stress-induced behavioral despair, anxiety or cognition, although it did affect metabolism. These findings suggest that ketamine might regain HPA axis function and lower cortisol, and present a unique way of protecting against stress-induced dysfunction developing in the first place. This could present patients with a novel opportunity of a rapid-onset period of decreased stress load and stress-induced pathology, which might aid in the facilitation of lasting cognitive-emotional and lifestyle changes.

### 2.2. Chronic stress activates microglia and initiates inflammatory processes

It has been suggested that hallmark characteristics of depression can be caused by the effect of chronic stress on microglia, the resident immune cells of the brain (Vanderzwaag et al., 2023). Microglia respond by releasing proinflammatory cytokines, which increase the permeability of the blood–brain barrier (BBB), whose purpose is to protect the brain from infiltration by toxic substances. This allows for an influx of peripheral cytokines and macrophages into the brain (Espey et al., 1997), where they can exert toxic effects on stress-sensitive and stress-mediating areas, such as the anterior cingulate cortex (ACC), amygdala, prefrontal cortex (PFC), and hippocampus (McEwen and Gianaros, 2010; Kim and Won, 2017; Vanderzwaag et al., 2023).

Evidence of causal links between inflammation and depression first emerged in the 1980s, when it was observed that a subset of patients given the proinflammatory cytokine interferon-α, developed depressive symptoms which subsided when treatment was discontinued (Renault et al., 1987). Interestingly, the patients seemed to develop different depression subtypes based on variations in dosage and lifestyle factors known to increase inflammation, prompting a line of investigation into how different manifestations of depression might result from different constellations of inflammatory challenges. Since then, the common inflammatory marker C-reactive protein (CRP) has been linked to both depression as a whole, and to specific symptoms such as fatigue, abnormal appetite, and cognitive dysfunction (Chang et al., 2012; Moriarity et al., 2023). Measurements of cerebrospinal fluid (CSF) have revealed associations between elevated levels of tumor necrosis factor (TNF) and reduced motivation, as well as elevated interleukin (IL)-6 and anhedonia (Felger et al., 2020). Furthermore, the CSF inflammatory markers correlated with high plasma CRP, as well as the severity of depressive symptoms. While these findings hint at associations between specific manifestations of depression and certain inflammatory markers, inflammatory reactions are a collaborative effort of a plethora of cytokines, and as such, it can prove challenging to predict targeting of specific symptoms by specific cytokines. On the other hand, if depression involves large-scale inflammation, the reduction of certain aspects of it may have downstream effects on other inflammatory processes and depressive symptomatology. Indeed, depression was recently characterized as a pro-inflammatory state in a large meta-analysis which found robust elevations of CRP, and proinflammatory cytokines IL-6, IL-3, IL-6, IL-12, IL-18, soluble IL-2 receptor, and TNF-α (Osimo et al., 2020). As inflammation in depression has been suggested to be facilitated by environmental challenges such as lifestyle factors, illness, and psychosocial stress (Felger, 2018), inflammatory and depressive symptom load might be alleviated by targeting these factors.

### 2.3. Social and lifestyle factors impact stress-immune interactions

Researchers have reported prolonged elevations of inflammatory markers in individuals exposed to long-term stress, especially early adversity (Taylor et al., 2006; Danese et al., 2007, 2008; Kiecolt-Glaser et al., 2011; Miller and Cole, 2012). These studies show that factors such as low socioeconomic status, interpersonal stress, parental separation, maltreatment, and abuse predicts future elevations of CRP, IL-6, and/or TNF-α. This phenomenon was further elucidated by Miller and Cole (2010), who found that growing up in a harsh family climate was associated with a shift towards an increasingly proinflammatory phenotype. This involved a progressive disruption of cortisol’s ability to regulate inflammatory activity, and an increased IL-6 response to a bacterial challenge. Significant elevations of IL-6 have also been observed in response to daily stressors in individuals who experienced childhood abuse (Gouin et al., 2012). These findings suggest that while stress can cause a general increase in inflammation, it can also heighten immune responses specifically in response to stressors.

This neuro-inflammatory sensitization to adversity is further described in the social signal transduction theory of depression (Slavich and Irwin, 2014), which explores how the brain responds to social-environmental adversity by modulating systemic stress and immune activity, which can ultimately act on the brain to induce typical manifestations of depression such as sad mood, anhedonia, fatigue, social withdrawal, and psychomotor retardation. This involves signaling from cytokines, and from the vagus nerve, whose signals to the brain regulate mood, motivation, arousal, sensitivity to social threat, and motor activity. Slavich and Irwin (2014) explain how this neuro-inflammatory link allows for a “preparatory pathogen host defense” involving anticipatory mobilization of immune cells to sites of possible injury and infection – an adaptive response in the face of actual threat. However, these social signal transduction pathways can be activated by the psychological experience of threat even when there is none; this can cause self-promoted long-term engagement of these systems, which is further worsened by neuro-inflammatory sensitization (Dhabhar, 2009; Slavich and Cole, 2013). This is further explored in social safety theory, which describes the implications of how the brain and immune system evolved to keep the body biologically and physically safe by ensuring social bonds and mounting anticipatory biobehavioral responses to social, physical, and microbial threats (Slavich, 2020). Importantly, negatively biased perceptions based on previous adversity can over-activate and prolong these biobehavioral responses, facilitating depression (Glaser and Kiecolt-Glaser, 2005; Furman et al., 2019; Slavich, 2020).

Neuroprogressive worsening is further facilitated by lifestyle factors associated with both depression risk and depressive behavior, such as physical inactivity, inflammatory diets, and abnormal sleep patterns (Berk et al., 2013). As these are important considerations in optimizing treatment effects for depressed patients, and provide insight into the complex nature of depressive pathology, a brief overview of central lifestyle considerations will be provided. Firstly, increased engagement in sedentary behavior is associated with a range of negative health outcomes, including depression, inflammation, and adiposity (Park et al., 2020). These have been observed to create a self-perpetuating cycle in which inactivity and diet changes contribute to adiposity, which increases inflammation, which strengthens depression, which again, facilitates inactivity and diet changes (Shelton and Miller, 2011). Secondly, increased consumption of saturated and *trans* fats, refined sugar, and ultra-processed food can increase risk of chronic inflammation, depression, and neurodegeneration (Li et al., 2017, 2022; Ljungberg et al., 2020). It can also affect the microbiota-gut-brain axis which has been implicated in stress, inflammation, and depressive pathology (Mitrea et al., 2022). Thirdly, an increasing number of people suffer from insufficient sleep quality or quantity, which over time increase the risk of depression, neurologic disease, chronic pain, gastrointestinal problems, and cardiovascular disease (Taylor et al., 2007; Buysse et al., 2008; Nutt et al., 2008). Identified contributors to sleep disturbances include sedentary behavior and adiposity (Shochat, 2012), demonstrating how these risk factors tend to overlap and affect each other. Note that while these lifestyle factors can play important roles in the pathogenesis of depression and prolonged inflammation, they can also be behavioral manifestations of depression or immune challenges (Mac Giollabhui, 2021). Due to shared immuno-inflammatory pathways, both depression and immune challenges can manifest as “sickness behavior” like fatigue, anhedonia, cognitive dysfunction, psychomotor retardation, and social withdrawal (Maes et al., 2012). These behaviors might further increase stress, inflammation-and depression-related pathology. For example, in addition to decreasing stress resilience through inadequate social support, social isolation is associated with cognitive, affective, immune-, and neuroendocrine dysfunction, and loneliness, with more depressive symptoms, greater functional impairment, and disruptions of cardiovascular activation and sleep (Cacioppo et al., 2002; Ozbay et al., 2007; Hawkley and Cacioppo, 2010; Theeke, 2010). Adding to this, stress-induced neuroendocrine and cardiovascular dysfunction might in turn increase deleterious effects of social isolation (Grant et al., 2009). These findings illustrate how interlinked aspects of physiology and health are, and how the straining of certain biological systems can contribute to progressive disruption on a systemic level. As seen in depression, prolonged engagement of the stress and immune systems can lead to more stress, inflammation, stress-induced inflammation, and inflammation-induced stress. This presents the challenge of targeting a highly entangled and self-reinforcing pathology; It also provides opportunities of decreasing the pathology from several angles, and allows for multiple mechanisms of antidepressant action for drugs like ketamine, which seem to interfere with several points in these cycles.

### 2.4. Different lines of investigations into ketamine’s immunomodulatory effects

Ketamine’s immunomodulatory effects were first observed in surgical patients who received the drug with other general anesthetics, and was later confirmed in a meta-analysis which found ketamine to suppress post-operative IL-6 elevations (Dale et al., 2012). Some noteworthy findings from these studies are that IL-6 remained lower when assessed a week later (Roytblat et al., 1998), and that doses as small as 0.15 mg/kg was able to downregulate both IL-6 and TNF-α (Beilin et al., 2007). Ketamine’s attenuation of post-operative inflammation prompts the question of whether it might interfere with inflammatory processes necessary for healing. This was investigated in a review which concluded that the unique mechanisms of ketamine allows for attenuation of systemic hyperinflammation, without affecting local healing processes (Loix et al., 2011).

Later studies have investigated the immunomodulatory effects of ketamine specifically in relation to antidepressant efficacy. In the studies, most of which included treatment resistant subjects receiving one 0.5 mg/kg dose of ketamine, reductions of proinflammatory cytokines were confirmed while its relation to antidepressant response varied. While some demonstrated associations between response and reductions of TNF-α (Chen et al., 2018), and elevated baseline IL-6 (Yang et al., 2015), others failed to find any significant relation between cytokine levels and response (Kiraly et al., 2017). Interestingly, changes in IL-8 levels have been significantly linked with response through its association with sex, where improvement in females was associated with reductions, while improvement in males was associated with elevations of IL-8 (Kruse et al., 2021). The cytokine was implicated again in a recent study, which found ketamine to decrease the IL-8–IL-10 ratio in their depressed subsample (Langhein et al., 2022). Baseline levels of the proinflammatory IL-8 relative to the anti-inflammatory IL-10 was higher in the depressed group and correlated with measures of neurodegeneration, while worse white matter integrity at baseline was associated with antidepressant response.

Few studies have investigated the lasting immunomodulatory effects of multiple doses of ketamine, although one did administer 6 doses over 12 days, and measured effects for 2 weeks after the final infusion (Zhan et al., 2020). Subjects obtained a response rate of 58.3% and a remission rate of 41.7%, which correlated with cytokine reduction on day one, but not at 2 weeks. Response was reliably predicted by lower baseline levels of the chemokine interferon-inducible T cell alpha chemoattractant (ITAC), which is suggested to have neuroprotective effects on stress and inflammation in depression (Miller, 2010). Data from the study by Zhan et al. (2020) was also used to explore cytokines in relation to hippocampal volume (Zhou et al., 2020) and comorbid pain (Zhou et al., 2021). Ketamine slightly increased hippocampal volume, but this was not significantly correlated with cytokine reduction at day 1. On day 14, there was a weak association between alterations of cytokines and tryptophan metabolites. In the study investigating comorbid pain, IL-6 reductions correlated with symptom reduction on day 1, but only in those with comorbid pain. As those suffering from physical pain in addition to depression responded more than controls and depressed without pain, this might indicate a further degeneration and alteration by ketamine of the ACC projections involved in mental and physical pain. It might also support previous findings suggesting that involvement of the opioid system and the analgesia caused by it is necessary for ketamine’s antidepressant effects (Klein et al., 2020).

Rodent studies have observed the effects of ketamine on stress-induced inflammation and depressive behavior in more controlled settings. Studies administering one 10 mg/kg dose have found ketamine to decrease stress-induced elevations of IL-6 and IL-1β in the hippocampus and PFC (Yang et al., 2013; Wang et al., 2015). Considering that inflammation-induced dysfunction in these regions are implicated in depression, this could provide translational value for understanding *in vivo* alterations of neuroinflammation by ketamine. Finally, others have subjected rats to injuries causing neuropathic pain, leading to some developing a depressive phenotype and lasting cytokine elevations (Xie et al., 2017). A 20 mg/kg dose of ketamine effectively reversed the depressive phenotype and serum concentrations of IL-6 and IL-1β, whose levels at baseline correlated with increased sucrose preference after ketamine. This might indicate that ketamine could reduce anhedonia through modulation of inflammatory processes which are compromising the reward circuitry. Taken together, these studies support previous findings of ketamine’s reduction of stress-induced depressive behavior and cytokine elevations, and show how ketamine can alter both systemic hyperinflammation, and neuroinflammation in key regions of depressive pathology.

### 2.5. Altered function of neural regions and networks implicated In depression

#### 2.5.1. Connectivity analyses linking inflammatory markers to symptomatology

Studies linking aberrant neural connectivity to elevated inflammation, shed light on how inflammation might alter brain function towards depressive states. Connectivity analyses show significant correlations of high CRP with decreased ventral striatum–ventromedial PFC connectivity, which in turn correlated with increased anhedonia (Felger et al., 2016). This links elevated immune markers to abnormal communication between the subcortical areas encoding reward, and the cortical areas playing key roles in cognitive-emotional, social, and self-referential processing. Furthermore, psychomotor retardation correlated with increased CRP through decreased dorsal striatum– ventromedial PFC connectivity, confirming abnormal communication between movement-initiating areas and the cortex. Additional analyses revealed a negative correlation between striatum– ventromedial PFC connectivity and levels of plasma IL-1β, IL-6, and IL-1 receptor antagonist, suggesting a linear relationship between circulating cytokines and corticostriatal connectivity. Similarly, worse performance in psychomotor speed tasks have been linked to increased levels of the proinflammatory cytokine IL-6, while better outcomes were associated with the anti-inflammatory cytokine IL-10 (Goldsmith et al., 2016). Elevated IL-6 has also been implicated in depressive memory deficits (Grassi-Oliveira et al., 2011), and has been identified as a mediator linking adiposity with cognitive dysfunction (Mac Giollabhui et al., 2020). These findings elucidate the possible effects of stress-induced inflammation on brain function and connectivity, in addition to identifying a potential proinflammatory mediator of the relationship between lifestyle factors and neurocognitive outcomes.

#### 2.5.2. Amygdala, hippocampus, and the “anti-reward network”

Prolonged stress causing HPA axis dysfunction and cortisol hyperactivity has been shown to lead to structural and functional disruption in the amygdala and hippocampus, key regions for facilitating physiological and behavioral responses to stress (Gray and Bingaman, 1996; MacQueen and Frodl, 2010; Zhang et al., 2018). Depressed individuals exhibit higher resting-state amygdala activity, and its reactivity to emotional and negative stimuli is shown to correlate with symptom severity, and with anxiety and anhedonia in those exposed to negative life events (Hamilton and Gotlib, 2008; Grogans et al., 2022; Hu et al., 2022). Greater neural sensitivity during encoding of negative stimuli has been attributed to increased baseline activity in the amygdala, and its increased functional connectivity with the hippocampus and dorsal striatum in depression (Hamilton and Gotlib, 2008). The hippocampus is known to be implicated in depression and crucial for memory (MacQueen and Frodl, 2010). The structure is vulnerable to damaging effects of chronic stress (Watanabe et al., 1992b; MacQueen and Frodl, 2010), exhibiting prolonged downregulation of GR mRNA levels and decreased GR expression (Kitraki et al., 1999). Other rodent studies supplement these findings by demonstrating alterations of amygdala–hippocampus projections thought to mediate anxious and social behavior (Liu et al., 2014; Qin et al., 2022). This involves disruption of the inhibitory gamma-aminobutyric acid (GABA) neurons responsible for regulating the projection, causing disinhibition and increased anxiety (Qin et al., 2022). Stress-induced dysfunction has also been demonstrated in amygdalar GABAa receptors, which the authors attributed to the upregulated glucocorticoids’ activation of GRs (Liu et al., 2014). The direct effect of glucocorticoids on the amygdala has been demonstrated by surgical implantations of corticosterone, which induced anxiety-like behavior and colonic distress – effects that were attenuated by administration of a CRH antagonist (Myers et al., 2005).

Ketamine has been shown to reduce amygdala–hippocampus reactivity to emotional stimuli, with reduced amygdala reactivity to negative stimuli correlating with resting-state connectivity to the pregenual anterior cingulate cortex (Scheidegger et al., 2016). A noteworthy finding from this study is that reduced neural reactivity to negative stimuli was predicted by the intensity of psychedelic alterations of consciousness. Ketamine has also been shown to improve hippocampal neurogenesis and learning in mice subjected to traumatic brain injuries, although it was also found to temporarily reduce neurogenesis and to increase microglial cell proliferation (Peters et al., 2018). While hippocampal atrophy has been associated with resistance to conventional antidepressants, a small study on humans found that smaller left hippocampus volume correlated strongly, although not significantly, with symptom reduction in response to ketamine (Abdallah et al., 2015).

The importance of managing disruptions in the hippocampus and amygdala is not only evident by their individual importance for everyday functioning, but also the fact that they are nodes in multiple neural networks which coordinate biobehavioral adaptation. An example is the “anti-reward network” arising from the habenula, a small structure in the epithalamus. It has been implicated in depression and suicidality, and its coordination with regions including amygdala and hippocampus has been suggested to cause large releases of CRH, norepinephrine, and dynorphin facilitating negative affect and anhedonia (Elman et al., 2013). The lateral habenula has been shown to be altered by ketamine, which acutely diminished its hyperactivity in depression-induced rodents (Malinow and Klein, 2020). It also improved learned helplessness and motivation, which could be suggestive of a shift of negative bias in outcome monitoring associated with depression. A study on monkeys demonstrated the lateral habenula to aid in outcome monitoring through detecting negative outcomes in real time, together with ACC, which maintained a representation of accumulated past experience (Kawai et al., 2015). The disruption of these regions in depression could help explain why patients feel unable to act in a motivated and confident manner to obtain their goals, making their alterations by ketamine an exciting avenue of research to follow in the coming years.

#### 2.5.3. The cingulate cortex and connected networks

The cingulate cortex is heavily implicated in depression and ketamine’s antidepressant actions, specifically its anterior and posterior sections (Alexander et al., 2021). The ACC plays critical roles in cognition and emotion, is implicated in depressive rumination and clinical pain (Alexander et al., 2021; Loggia et al., 2013), and has extensive connections with multiple cortical and subcortical structures including the amygdala and hippocampus (Stevens et al., 2011). Its projections allow it to integrate information about expected value, cost, reward and punishment, and to facilitate context-appropriate behavioral responses (Rolls, 2019). With this in mind, it is perhaps not surprising that depressed individuals exhibit abnormal metabolic activity and simultaneous gray matter reductions reflective of glial atrophy in the subgenual ACC (sgACC; Drevets et al., 2008b). Researchers have also found high sgACC activity to correlate with suicidal ideation in treatment resistant patients, and ketamine’s reduction of sgACC activity to correlate with reduced suicidality (Ballard et al., 2014). Ketamine has also been shown to reduce depressive symptoms through increasing functional connectivity between the sgACC and the right lateral PFC (Gärtner et al., 2019). All responders had low connectivity at baseline, which is congruent with previous suggestions that low sgACC– dorsolateral PFC (dlPFC) connectivity could be a biomarker for depression (Benschop et al., 2022), and that sgACC–left dlPFC connectivity could be a marker of antidepressant response (Baeken et al., 2014). The sgACC also displays increased connectivity with the hippocampus in depressed patients, which has been linked to increased anhedonia (Morris et al., 2020). Although ketamine did not decrease this hyperconnectivity, it improved depressive symptoms including anhedonia (Morris et al., 2020).

The ACC was recently suggested to be a key locus of ketamine’s antidepressant actions through its alterations of two main networks implicated in depression (Alexander et al., 2021). First, ketamine blocks a “medial emotional pain network,” consisting of different components of the ACC, insula, and thalamus. This hypothesis builds on that of Opler et al. (2016), who proposed a multi-step process where ketamine (a) produces acute suppression of firing in circuits representing mental anguish, “breaking their vicious cycle”; and (b) strengthens dorsolateral dlPFC circuits that regulate these pain pathways, a process likely involving increased prefrontal synaptogenesis. The second hypothesized mechanism of action is modulation of activity in the default mode network (DMN), which includes pregenual ACC, medial PFC, dorsomedial PFC, the posterior cingulate cortex, and hippocampus (Alexander et al., 2021). Activity in the DMN is elevated when focus is internally rather than externally directed, and declines during and after goal-directed activities that are not self-referential in nature (Watkins and Roberts, 2020). The DMN’s functional connectivity with the sgACC seems to be a neural substrate for depressive rumination, reflecting the integration of self-referential signals from the DMN and affective behavioral withdrawal signals from the sgACC (Hamilton et al., 2015). Rumination is predictive of relapse to depression (Ronold et al., 2020), exacerbation of pain (Staniszewski et al., 2023), greater depressive symptom burden and impairment including cognitive and interpersonal difficulties, and might also predict anxiety, substance abuse, eating disorders and self-harm (Nolen-Hoeksema et al., 2008). Furthermore, rather than leading to active problem solving, rumination involves a recurrent passive focus on problems and emotions related to them, often without leading to any action. It is therefore of critical importance to interrupt the ruminative way depressed individuals think about themselves and their problems, and strengthen their problem solving abilities (Nolen-Hoeksema et al., 2008). By attenuating behavioral withdrawal signals based on assumed negative outcomes possibly overrepresented in the ACC, and reducing hyperactivation of anxiety and stress-provoking projections, ketamine might aid in facilitating behavioral action. Although research is lacking on how long ketamine’s direct effects can be expected to last, the acute and post-acute lifting of depressive thought patterns might facilitate hope and a belief in the way forward, a powerful tool for combating the learned helplessness and pessimism afflicting depressed individuals and preventing them from taking actions to better their health.

## 3. Synaptogenesis and degeneration – a balance of toxicity and protection

**Figure 3:**
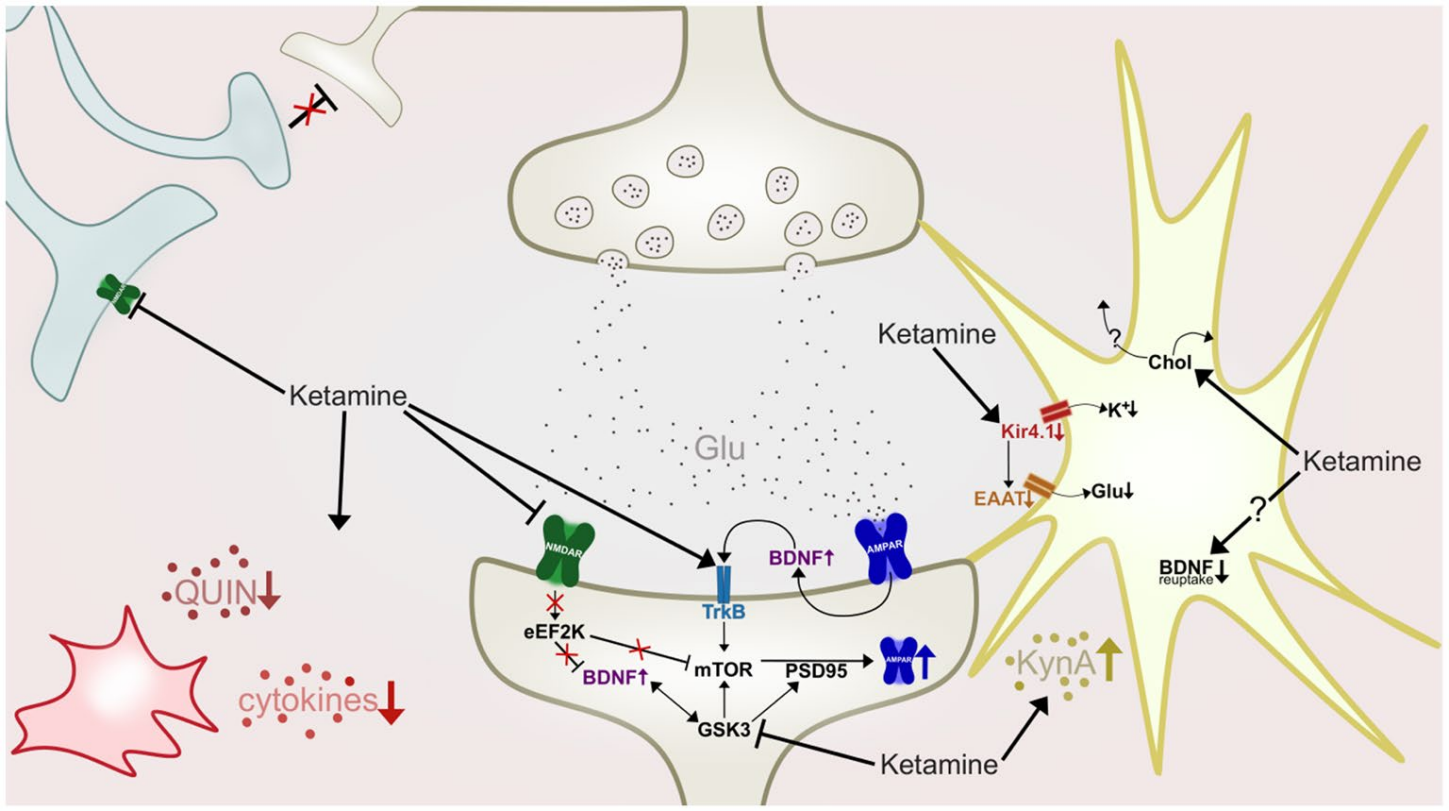
A highly simplified illustration of some of ketamine’s hypothesized antidepressant mechanisms of action at an inter-and intraneuronal level. Ketamine antagonize NMDARs (green) of GABAergic interneurons (light blue), causing disinhibition of pyramidal neurons (beige, top), resulting in a glutamate (Glu) surge. Activation of postsynaptic AMPAR (dark blue) initiate intracellular cascades which in turn increase synaptic BDNF levels and TrkB activation. Ketamine also antagonizes post-synaptic NMDARs, deactivating eEF2K, leading to desuppression of BDNF and mTOR. Increased mTOR phosphorylation leads to increases in levels of PSD-95 and AMPAR subunits. GSK-3 is inhibited by ketamine, resulting in increased mTOR and PSD-95 activity. In the astrocyte (yellow) ketamine downregulates activity of the Kir4.1 channel, causing less influx of potassium (K+), depolarization of the cell, and by consequence, reduction of EAAT activity and reduced retrieval of glutamate from the synapse. Ketamine also increase cholesterol (Chol) density in the astroglial membrane, which is hypothesized to cause a flux of cholesterol to nearby neurons. Astrocytes are also shown to produce and release more kynurenic acid (KynA) after ketamine treatment, and could also have impaired BDNF reuptake ability as ketamine stabilizes astrocytic fusion pores in a narrow configuration. Ketamine also affects microglia (red) and inflammation, attenuating hyperactivity of proinflammatory cytokines, and reducing levels of quinolinic acid (QUIN).

In the recent decade much research has focused on elucidating mechanisms underlying ketamine’s potential for aiding in the regulation of synaptic plasticity and neuronal plasticity. The prevailing hypothesis of ketamine’s contribution to restoring the brain’s synaptogenic capacities, is through NMDAR antagonism dependent enhancement of α-amino-3-hydroxy-5-methyl-4-isoxazolepropionic acid receptor (AMPAR) functioning (Zarate and Machado-Vieira, 2016). This is observed to, at least partly, be achieved by a preferential antagonism of NMDAR on GABAergic interneurons (Cohen et al., 2015). The NMDAR antagonism blocks these interneurons from applying their inhibitory input to cortical pyramidal neurons (Sanacora and Schatzberg, 2015), which in turn leads to what some authors refer to as a “glutamate surge” from these pyramidal neurons, i.e., a large and rapid excretion of glutamate (McMillan and Muthukumaraswamy, 2020). This glutamate surge causes activation of postsynaptic AMPAR and consequent depolarization of the postsynaptic cell, thus leading to an initiation of intracellular cascades and synaptogenesis (Duman et al., 2012; Aleksandrova et al., 2017). As the research on ketamine’s involvement in enhancing synaptogenesis has progressed, a set of diverse, but not mutually exclusive mechanisms have been identified (Figure 3), that may act synergistically to induce a sustained strengthening of excitatory synapses (e.g., Lener et al., 2017; McMillan and Muthukumaraswamy, 2020; Stenovec et al., 2020b). One of the mechanisms implicated as central to ketamine’s antidepressant mechanisms, is a rapid elevation of the growth factor brain-derived neurotrophic factor (BDNF), also implicated in the pathophysiology of depression and response to conventional antidepressants (Björkholm and Monteggia, 2016).

### 3.1. BDNF

Duman and Li (2012) reviewed the evidence for disruption of important growth factor systems in depression, and concluded that there is strong evidence supporting that reduced levels of growth factors, BDNF included, are associated with loss of neurons and glia in stress-and depression-related disorders. The first evidence linking BDNF to antidepressive effects came from studies showing that antidepressant treatment enhanced BDNF and tropomyosin receptor kinase B (TrkB) mRNA expression in the hippocampus and cortical regions of rodents, which coincided with the timeframe of the onset of antidepressant effects (Nibuya et al., 1995, 1996). TrkB is a receptor for BDNF (Klein et al., 1991), and it has been shown that activation of this receptor is a critical mediator of activity-dependent synaptic plasticity (Park and Poo, 2013). Later findings have further elucidated BDNF’s implication in antidepressant response, demonstrating that loss of BDNF in the dentate gyrus of the hippocampus attenuated the antidepressant effects of SSRIs and tricyclic antidepressants (Adachi et al., 2008). The implication of dysregulated BDNF in depression is also supported by both human post-mortem (Duman and Li, 2012) and rodent (McEwen, 2007; Castrén and Rantamäki, 2010) studies, showing that human depression and rodent models of depression are associated with decreased expression of several neurotrophic factors, including BDNF. Blood serum analyses of depressed patients also show reduced BDNF levels before treatment, but additionally that these reduced levels can be rescued by antidepressant treatments, supporting that BDNF is a biomarker of depression and treatment response (Sen et al., 2008; Bocchio-Chiavetto et al., 2010). Both SSRIs and tricyclic antidepressants have been demonstrated to increase BDNF expression, but only after chronic administration over several weeks and not after acute treatment (Nibuya et al., 1995, 1996). While tricyclic antidepressants have been demonstrated to protect against stress-induced dendritic retraction if administered prior to stressful stimuli (Watanabe et al., 1992a), there has generally been a lack of evidence of conventional antidepressants reversing stress induced atrophy (Duman and Li, 2012). A possible explanation for this is that conventional antidepressant treatment only seems to upregulate BDNF expression, but not BDNF release in itself, which is required for synaptogenesis (Chen et al., 2006; Jourdi et al., 2009; Hoeffer and Klann, 2010).

Contrary to conventional antidepressants, ketamine induces a rapid increase in BDNF protein levels (Autry et al., 2011; Haile et al., 2014). Responders to ketamine treatment had elevated plasma BDNF levels at 240 min post infusion (Haile et al., 2014), while a rodent study demonstrated elevated BDNF levels at 30 min after treatment (Autry et al., 2011). It has also been shown that successful antidepressant ketamine treatment increases BDNF levels in the hippocampus of rodents (Garcia et al., 2008), and that the antidepressant effects of ketamine are attenuated in mice with specific BDNF-and conditional TrkB-knockout (Autry et al., 2011). A recent review concluded that the TrkB receptor is an important mediator of antidepressant drug actions (Casarotto et al., 2022), and it has also been reported to reactivate a state of juvenile-like plasticity in the adult brain (Umemori et al., 2018). Interestingly, both conventional antidepressants, such as SSRIs and tricyclic antidepressants, and ketamine has been shown to directly bind to TrkB (Zanos et al., 2016). These findings indicate that BNDF and TrkB are crucial components in the response to ketamine and other antidepressants.

The discovery of a BDNF single nucleotide polymorphism (SNP) that leads to decreased processing and activity-dependent release of BDNF, namely Val66Met, has further aided in elucidating the significance of BDNF in depression and response to ketamine. Studies on mice expressing the human Val66Met SNP have revealed that these mice show an attenuated antidepressant response to ketamine treatment (Liu et al., 2012). This is further supported by a small human trial that showed an increased response to ketamine treatment in the patients not expressing the Val66Met SNP versus those expressing it (Laje et al., 2012). This SNP is not directly associated with depression, but the BDNF gene polymorphism has been shown to increase vulnerability to develop depression for individuals exposed to early life stress or trauma (Kaufman et al., 2006; Kim et al., 2007; Gatt et al., 2009). Additionally, the Val66Met SNP was associated with increased inflammation, increased neurodegeneration, apoptosis, and activation of microglia in the hippocampus and cortex after a traumatic brain injury, possibly reflecting susceptibility linked to reduced BDNF levels (Giarratana et al., 2019). Further supporting the importance of BDNF in antidepressant response, the Val66Met as well as two other BDNF SNPs have been associated with an attenuated response to conventional antidepressant treatment in a human clinical trial (Kocabas et al., 2011).

It has also been hypothesized that ketamine could induce microglial release of BDNF (Vanderzwaag et al., 2023). Glebov et al. (2015) found that serotonin agonism of 5-HT2A/2B/4 receptors in mice cause the release of microglial exosomes, membranous vesicles that contain different proteins and lipids prepared for extracellular release. A review considering the findings above, together with findings that such exosomes play a critical role in communication between neurons, astrocytes, and, microglia (Paolicelli et al., 2019), has led to the formulation of a hypothesis that 5-HT22A/2B/4 receptor agonism via ketamine or classical psychedelics induce a wave of exosomal release (Vanderzwaag et al., 2023). Vanderzwaag et al. (2023) highlight that if these microglial exosomes contain BDNF or insulin-like growth factor-1, it could be an avenue to explain the rapid neuroplastic effect on synaptic density reported in ketamine and psychedelic treatment.

### 3.2. Eukaryotic elongation factor 2 kinase

Investigations into ketamine’s mechanisms of action has revealed that while the preferential antagonism of NMDARs on GABAergic interneurons trigger a surge of glutamate and subsequent AMPAR activation, ketamine also antagonizes postsynaptic NMDARs, found to be associated with the increase in BDNF observed after ketamine treatment. As the postsynaptic NMDARs are blocked, this deactivates the eukaryotic elongation factor 2 kinase (eEF2K), stopping a signaling cascade that would otherwise suppress BDNF (Autry et al., 2011). Subsequent work utilizing mice with eEF2K-knockout did not show an antidepressant response to ketamine, or any increase in BDNF protein expression as a response to its administration in these mice, contrary to the control group (Nosyreva et al., 2013). It has also been proposed that eEF2K-inhibiton reduces suppression of another important protein involved in the induction of synaptogenesis and ketamine’s mechanisms, namely the mammalian target of rapamycin (mTOR; Hoeffer and Klann, 2010; Li et al., 2011; Björkholm and Monteggia, 2016).

### 3.3. Mammalian target of rapamycin

Ketamine has been shown to transiently increase mTOR phosphorylation, which was further associated with an increase in levels of synapsin-1, postsynaptic density protein-95 (PSD-95) and GluR1 (Li et al., 2011), all important for the formation, maturation and function of new spine synapses (Hoeffer and Klann, 2010). These changes were associated with an enhancement of synaptic function, through increased number and maturation of dendrite spines of pyramidal neurons, and it was further shown that administration of an mTOR inhibitor abrogated these synaptogenic effects (Li et al., 2011). Other antidepressant modalities such as ECT, imipramine, or fluoxetine, did not significantly influence mTOR signaling in Li et al. (2011) study.

### 3.4. Glycogen synthase kinase-3

Another factor implicated in ketamine’s antidepressant efficacy, as well as mTOR regulation, is glycogen synthase kinase-3 (GSK-3), a widely influential enzyme, playing a key role in biological processes such as oxidative stress, inflammation and ultimately neurogenesis (Jope, 2011). Dysregulation of GSK-3 has been associated with several neuropsychiatric disorders, including depression, and has been observed to have several detrimental effects, potentially impairing neuroplasticity, neurogenesis, gene expression, and the capability of neurons to endure and respond to stressful conditions (Jope and Roh, 2006). As reviewed by Jope (2011), a number of links have been identified between abnormal GSK3 activity and susceptibility to depression. Inhibition of this molecule modulates mTOR signaling, and it has been noted that GSK3 dysregulation may occur due to depression-associated deficiency in certain neuromodulators that otherwise would inhibit this kinase (Jope, 2011). It has also been shown that the deficient inhibition of GSK3 can be rescued by lithium treatment (Klein and Melton, 1996).

Additionally, it has been demonstrated that ketamine can induce GSK-3 inhibition, and further that the increases in BDNF levels, mTOR phosphorylation, and AMPAR signaling enhancement seen after ketamine treatment was dependent on this inhibition (Beurel et al., 2011, 2016). Further support for the implication of GSK-3 in antidepressant effects is found in reports showing that co-administration of the GSK-3 inhibitor lithium, together with ketamine, lowered the dose of ketamine needed to provide antidepressant effects in mice (Liu et al., 2013; Chiu et al., 2014; Viana et al., 2021). As the final part of their study, Beurel et al. (2016) showed that GSK-3 inhibition by ketamine reduced phosphorylation of PSD-95, thus diminishing the internalization of AMPA GluA1 subunits (i.e., stabilizing them on the surface). It is noteworthy that GSK-3 uses PSD-95 as a substrate, and that PSD-95 is regulating AMPA receptor trafficking (Nelson et al., 2013). Johnson-Farley et al. (2006) demonstrated that BDNF inhibits the activity of GSK-3 in the hippocampus, leading to Björkholm and Monteggia (2016) hypothesizing that GSK-3 inhibition is a downstream consequence of ketamine’s antidepressant mechanisms, rather than initiatory.

### 3.5. Relevance of astrocytes in the antidepressant mechanisms of ketamine

Astrocytes are both recipients and excreters of BDNF (Autry and Monteggia, 2012; Parkhurst et al., 2013; Björkholm and Monteggia, 2016; Hisaoka-Nakashima et al., 2016), and several lines of research suggest modulation of astroglial physiology as an antidepressant mechanism of ketamine (Mitterauer, 2012; Lasič et al., 2016, 2019; Stenovec et al., 2020a). This is promising as their dysfunction has been linked to a range of psychiatric and neurodegenerative diseases, including depression, and some have argued that dysfunction in astrocytes may be implicated in all neurological, neuropsychiatric and neurodegenerative disease (Verkhratsky et al., 2014; Pekny et al., 2016). Astrocytes are the most functionally diverse of the glial cells, and are among many things capable of producing and releasing cytokines (Aloisi et al., 1992; Meeuwsen et al., 2003), maintaining the integrity of the blood–brain-barrier, providing structural support to neurons, as well as various metabolic functions like storage of glycogen, and uptake of glutamate and ions from the synaptic cleft (Volterra et al., 2014). Importantly, there is an observed prevalence of astroglial atrophy and degeneration in depression (Sofroniew and Vinters, 2010; Rajkowska and Stockmeier, 2013; Verkhratsky et al., 2014). A post-mortem study of depressed subjects, observed reduced astroglial density, but only in the population of subjects who had not received any antidepressant treatment (Cobb et al., 2016). The astroglial atrophy observed in depression further reduces brain resilience, capacity for change and growth through several astrocyte-associated processes.

#### 3.5.1. Glutamate excitotoxicity, astrocytes, and ketamine

One of astrocytes’ critical roles is protecting synaptic structures against excitotoxic effects through modulating glutamate homeostasis, regulating its release, uptake and metabolism (Anderson and Swanson, 2000). Glutamate has been implicated as a major source of oxidative stress in the brain since the early 1990s (Coyle and Puttfarcken, 1993), and glutamate-associated NMDAR excitotoxicity was already implicated as a target for antidepressant therapies in 1990 (Trullas and Skolnick, 1990). Astrocytic importance in glutamate homeostasis is supported in reports showing that neuronal vulnerability to glutamate toxicity is 100-fold greater in astrocyte-poor cell cultures, compared to cell cultures abundant in astrocytes (Rosenberg et al., 1992). Glutamate reuptake into astrocytes occurs through the glutamate transporters amino excitatory amino acid transporter-1 and 2 (EAAT1 and EAAT2; Furuta et al., 2005), with these transporters appearing to be responsible for 80–90% of extracellular glutamate reuptake (Mahmoud et al., 2019). These glutamate transporters appear to be dysregulated in depression, as postmortem brain samples from depressed subjects show reduced expression of mRNA for EAAT1 and EAAT2 in the ACC and dorsolateral PFC (Choudary et al., 2005).

Several studies have demonstrated ketamine’s ability to regulate astrocytic glutamate reuptake, through downregulating the expression and density of a type of inward rectifying potassium channel called Kir4.1 (Stenovec et al., 2020a; Frizzo and Ohno, 2021), which has been reported to be upregulated in depression (Xiong et al., 2019). This channel is a major conduit for the movement of potassium between the extracellular space and the astrocyte (Nwaobi et al., 2016; Breslin et al., 2018), and it has been shown that the efficiency of EAATs are dependent on astrocyte membrane hyperpolarization (Olsen and Sontheimer, 2008; Bay and Butt, 2012). It was also demonstrated that ketamine reduce the expression and density of the Kir4.1 channels, which leads to depolarization of astrocyte membranes, reduced EAAT activity, and consequently reduced uptake of glutamate and potassium into astrocytes (Frizzo and Ohno, 2021). Disturbance in Kir4.1 function and potassium transport has also been linked to abnormal firing patterns in the lateral habenula, which is further associated with a depression-like phenotype at the behavioral level (Cui et al., 2018; Yang et al., 2018). Yang et al. (2018) found that ketamine administration attenuated Kir4.1 overactivity and recovered the depressive-like behavior. Stenovec et al. (2020a) further elucidated the mechanisms behind ketamine’s Kir4.1 modulation, showing that ketamine induced a reduction in trafficking of these channels to the astrocyte membrane.

#### 3.5.2. The kynurenine pathway

While ketamine induce a strong glutamate release into the synapse and blocks its reuptake, it also blocks post-synaptic NMDARs (Autry et al., 2011), and can thus protect against potential excitotoxicity from glutamatergic NMDA-agonism. This protective mechanism is also relevant for another endogenous NMDA-agonist, namely quinolinic acid (QUIN) of the kynurenine pathway. Being a glutamatergic agonist acting on NMDAR, QUIN exerts similar neurotoxic effects as glutamate, considered to eventually involve reactive oxygen and nitrogen species (Tavares et al., 2002; Meier et al., 2016). QUIN is an intermediary metabolite in a major pathway for cell metabolism, through which tryptophan is converted via several steps into nicotinamide adenine dinucleotide (NAD+; Pérez-De La Cruz et al., 2007). NAD+ in an important coenzyme in generating cellular energy, and maintaining mitochondria health (Bender, 1983; Covarrubias et al., 2021). As the kynurenine pathway is critical in producing cellular energy, and sustaining the inflammatory process requires a major expenditure of energy, these two appear to function in close relationship to each other. It has been observed that the proinflammatory cytokines IL-1ß, IL-6, and TNF-α all upregulate the enzyme indoleamine-pyrrole 2,3-dioxygenase (IDO; Maes et al., 2007), which is the key factor in metabolizing tryptophan into kynurenine in the CNS and immune system (Guillemin et al., 2001).

In light of the elevated inflammation associated with depression, it is not surprising that the kynurenine pathway also appears to be dysregulated in several studies on depressed patients (Ogyu et al., 2018; Kadriu et al., 2019). The metabolism of tryptophan into kynurenine in the CNS occurs under homeostatic conditions primarily in astrocytes (Guillemin et al., 2001) and microglia (Heyes et al., 1996; Espey et al., 1997). However, an important inflammatory process linked to a disruption in kynurenine pathway metabolism towards neurotoxicity, is the gradual breakdown of BBB-integrity, enabling an influx of cytokines and macrophages from peripheral tissue from outside the CNS (Espey et al., 1997). Microglia and the infiltrating macrophages appear to be responsible for metabolism towards the neurotoxic QUIN and NAD+ (Guillemin et al., 2001). Macrophages appear to have around a 32-fold greater capacity for synthesizing QUIN compared to microglia, thus increased BBB-permeability may be a central factor causing KYN pathway dysregulation in favor of neurotoxicity (Espey et al., 1997; Guillemin et al., 2001; Parrott et al., 2016; Ogyu et al., 2018). Additionally, the observed atrophy of astrocytes in MDD could further exacerbate kynurenine pathway dysregulation, due to them being involved in synthesizing kynurenic acid (Guillemin et al., 2001) Kynurenic acid is another kynurenine pathway metabolite, generally considered neuroprotective, partly because of its function as NMDA-antagonist (Foster et al., 1984; Savitz, 2020). This dynamic is supported by evidence showing that MDD subjects generally are characterized by reduced levels of kynurenic acid (Ogyu et al., 2018; Kadriu et al., 2019). These studies also revealed that the patients who had not received any form of antidepressant treatment showed elevated QUIN levels. As reviewed so far, inflammation appear to induce KYN pathway dysregulation through at least three distinct mechanisms, (a) upregulation of IDO activity by proinflammatory cytokines, (b) increasing BBB-permeability, thus enabling the influx of macrophages that synthesize QUIN at a much greater rate, and (c) atrophy of astrocytes leading to less kynurenic acid synthesis.

Ketamine is shown to restore kynurenine dysregulation, with depressed patients exhibiting reduced levels of QUIN, and elevated levels of kynurenic acid after ketamine treatment (Ogyu et al., 2018; Kadriu et al., 2019). It is likely that ketamine contributes to this by modulating inflammation, reducing levels of proinflammatory cytokines (e.g., IL-1ß, IL-6, and TNF-α; Kadriu et al., 2019), which could further contribute to lessening BBB-permeability and restoring astrocytic functioning.

#### 3.5.3. Astrocytic cholesterol

One of the key synapse promoting factors synthesized and released by astroglia to neurons is cholesterol (Mauch et al., 2001), and modulation of astroglial cholesterol is implied as a part of ketamine’s antidepressant mechanisms (Lasič et al., 2016, 2019; Stenovec et al., 2020b). The loss of glial cell numbers reported in depression, suicide victims, and animal models of depression, has been hypothesized to be etiopathologically associated with insufficient supply of cholesterol from astrocytes to neurons (Rajkowska and Stockmeier, 2013; Sanacora and Banasr, 2013). Lipoprotein bound cholesterol is largely prevented from crossing the BBB (Björkhem and Meaney, 2004), and the brain is thus largely responsible for its own cholesterol production, which plays an important role in membrane fluidity, vesicle formation and synaptogenesis (Pfrieger and Ungerer, 2011). Cholesterol is an integral part of any membrane, and the delivery of astroglial-derived cholesterol to neurons has been reported to be the limiting factor regulating synapse formation (Dietschy and Turley, 2001; Ferris et al., 2017). Interestingly, the appearance of most synapses is temporally and spatially associated with the development of astrocytes, supporting the notion that astrocyte-derived cholesterol is indeed important for synapse formation (Ullian et al., 2001). This hypothesis is based on reports that neurons produce enough cholesterol themselves to survive and grow, but that the cholesterol requirements of synapses are too large for the neurons themselves to produce (Mauch et al., 2001). The CNS has a high demand of cholesterol, as synapses often have high numbers of postsynaptic spines, pre-synaptic vesicles with particularly high cholesterol contents (Takamori et al., 2006), and large areas of membranous surface (Goritz et al., 2005). As a consequence of these demands on CNS cholesterol production, it has been argued that MDD-associated loss of astroglial cell populations is causally linked to some of the functional abnormalities seen in depression (Theodosis et al., 2008). This is further supported by reports from rodent studies, where administration of cholesterol induced synapse growth, and enhanced their spontaneous and evoked activity within 3 days (Goritz et al., 2005).

The mechanisms behind ketamine’s increase in cholesterol still remains unclear (Stenovec et al., 2020a,b). However, some indications have come from two reports from Lasič et al. (2016, 2019), where they demonstrated a mechanism whereby ketamine induced a stable narrow configuration of astrocytic fusion pores, which was correlated with a reduced capacity for vesicle retrieval, indicating a fusion pore state incapable of transitioning to full vesicle fission (Lasič et al., 2016). They highlighted their finding as a novel mechanism behind ketamine’s antidepressant efficacy, one that might play a role in astroglial release and uptake of molecules, modulating synaptic activity. Ketamine’s effect on the fusion pores happened almost immediately after administration, and were long-lived, leading the authors to propose a hypothesis that the stabilization of the fusion pores in a narrow configuration might be a key part of ketamine’s antidepressant mechanisms, which may reset neural-glia networks. In a subsequent study, Lasič et al. (2019) demonstrated that ketamine increased the density of cholesterol rich domains in cell membranes of cultured rat astrocytes. Building on the discoveries of Lasič et al. (2016, 2019) and Stenovec et al. (2020b) hypothesize that the prevention of full vesicle retrieval by ketamine, hinders internalization of cholesterol from astrocyte membranes. Further they argue that ketamine-treated astrocytes with heightened membrane cholesterol content could exhibit a transient boost in cholesterol flux towards surrounding neurons, and that this could counteract the pathophysiological reduction in the number of synapses in the PFC observed in MDD subjects (Kang et al., 2012). Ketamine’s modulation of astrocytic vesicle fission (Lasič et al., 2016), could also impair BDNF reuptake into astroglia, resulting in increased extracellular levels of BDNF (Vignoli et al., 2016).

## 4. Discussion

This review has investigated the research on depressive pathology seen through a model of stress, inflammation, neurotoxicity, and neurodegeneration (Figure 1), and the ways in which ketamine is suggested to target key points in this self-reinforcing cycle (Figures 2,3). To summarize, ketamine acts on a system-and network level by regaining function and attenuating pathological hyperactivity; On the cellular and molecular level, ketamine initiates a critical physiological shift, facilitating neuronal and glial survival and proliferation. Rodent studies indicate that ketamine may increase stress resilience (Brachman et al., 2016), in addition to restoring HPA axis function and reducing elevated cortisol levels (Wang et al., 2019). Its rapid downregulation of exacerbated inflammation further reduces the impact of stress-induced immune dysfunction, leading to reduced levels of CNS inflammatory cytokines (Loix et al., 2011; De Kock et al., 2013). This attenuation of neuroinflammation could reduce neurotoxicity and neurodegeneration of brain areas whose dysfunction maintains depressive pathology. Furthermore, reduction of inflammation has downstream effects on the KYN pathway through decreasing IDO levels (Guillemin et al., 2001). Ketamine has also been shown to cause a shift in kynurenine metabolism, which involves reducing levels of neurotoxic QUIN, while increasing levels of neuroprotective kynurenic acid (Kadriu et al., 2019; Kopra et al., 2021). As kynurenic acid production is mainly the responsibility of astrocytes (Guillemin et al., 2001), the increase in the levels of this acid could reflect an enhancement of astrocytic function and density, which also has been implicated as a part of ketamine’s antidepressant mechanisms (Stenovec et al., 2020b). Ketamine is demonstrated to increase astroglial cholesterol content, possibly further aiding in synapse and membrane growth, maintenance and functioning (Lasič et al., 2016, 2019). Additionally, ketamine contributes to a decrease in surface density of Kir4.1 channels on astrocytes, aiding in both potassium and glutamate homeostasis, and optimal neuronal firing, associated with attenuation of depressive behavior (Stenovec et al., 2020a; Frizzo and Ohno, 2021). Through NMDAR-antagonism, ketamine blocks excitotoxicity exerted by glutamate and QUIN (Kadriu et al., 2019). Additionally, blocking NMDA-calcium influx inhibits eEF2K, leading to BDNF no longer being suppressed (Autry et al., 2011). Increased BDNF and modulation of mTOR and GSK-3 (Beurel et al., 2011, 2016) increases the number and maturity of AMPAR subunits on dendrites (Li et al., 2011), reflecting increased capacity for synaptogenesis. Furthermore, ketamine might increase synaptic BDNF through decreasing astrocytes’ capacity for vesicle retrieval (Lasič et al., 2016), and microglial BDNF release through stimulating 5HT2A/2B/4 receptors (Vanderzwaag et al., 2023). The increase in BDNF and related synaptogenic factors are considered essential to ketamine’s antidepressant effects (Li et al., 2011; Duman et al., 2016), and BDNF has been identified as a potential candidate as a biomarker of depression severity and treatment response (Sen et al., 2008; Bocchio-Chiavetto et al., 2010). By aiding neuronal integrity and function, ketamine may prevent maintenance or exacerbation of pathological mechanisms facilitated by network dysregulation, such as faulty appraisal initiating maladaptive threat responses. This involves strengthening the function of neural networks which regulate stress and signals reward, while attenuating hyperactivity in networks representing rumination, mental anguish and pain (Opler et al., 2016; Morris et al., 2020; Alexander et al., 2021). This may lead to decreased stress-induced pathology, and consequently, less inflammation, neurotoxicity, neurodegeneration, and depressive symptom load.

### 4.1. Limitations

A major limitation of the design is that narrative reviews are more prone to bias than systematic reviews. Whereas the latter can draw conclusions based on a systematically gathered, and more quantitative presentation of the available research, narrative reviews are based on a qualitative selection and presentation of studies which may have unintentionally omitted relevant findings. Consequently, narrative reviews are more prone to selection and reporting bias, and could be exacerbating the compromised validity already induced by publication bias and increased availability of positive findings over null findings.

The search for evidence outside of the model of stress, inflammation, neurotoxicity, and neurodegeneration was limited due to time restrictions and attempts to keep the review focused. For the same reasons, this review does not explore investigations of bipolar depression or ketamine’s enantiomers and metabolites. An inclusion of considerations from the literature on enantiomers and metabolites could have resulted in a more nuanced presentation of actions with indications of exactly what part of ketamine’s molecule or metabolic ladder the effects arise from.

A limitation of the included ketamine studies is that most do not assess effects over longer time courses, making ketamine’s long-term effects largely unexplored. Furthermore, the timing of assessments of response and remission in clinical trials could be another limitation to generalizability, as most measure symptomatology relatively shortly after administration. While symptoms must be present for 2 weeks for depression to be diagnosed, clinical studies establish remission hours or days after drug administration. By using stricter operationalizations of response, and particularly remission, studies could avoid exaggeration of effects. A general limitation of ketamine trials is that blinding is notoriously difficult with substances like ketamine, as patients tend to notice its effect or lack thereof, making saline an inadequate placebo (Blier et al., 2012). Another limitation we identified was that of lacking control of concomitant medication in studies. Several studies only employed ketamine as an add-on therapy in addition to conventional antidepressants, potentially confounding these results.

### 4.2. Implications and considerations for clinical implementation

#### 4.2.1. Dependence and safety

The rapid-acting mechanisms of ketamine allow it to be an antidepressant not intended for chronic use, presenting patients with the opportunity of improving without becoming dependent on the medication. In contrast, conventional antidepressants require daily administration over several weeks to take effect, and can cause dependence and severe adverse effects if discontinued (Horowitz et al., 2023). The acute effects of ketamine might minimize these risks. Ketamine is considered safe when administered in clinics (typically as a 0.5 mg/kg intravenous dose of racemic ketamine over 40 min), and common side effects such as dizziness, increased heart rate, dissociation and perceptual disturbances are transient (McIntyre et al., 2021). In their expert review, McIntyre and colleagues argue that there is no proven abuse potential when ketamine is administered as described, although they point out that early studies on opiates argued similarly. It must be noted that ketamine is a drug with abuse potential, and that this makes patients with a history of drug addiction likely to be excluded from research and treatment by clinicians. It should also be noted that some patients report that what they appreciated about the treatment was the reduction of depressive symptoms and that they would rather not receive the ketamine infusions if these benefits were maintained otherwise (Blier et al., 2012). Recently, a phase 2 clinical trial supported ketamine as a tool in the treatment of alcohol use disorder (Grabski et al., 2022), suggesting that ketamine’s potential for causing or treating substance abuse is highly context-dependent.

#### 4.2.2. Considerations for optimizing and maintaining treatment efficacy

Ketamine used in combination with psychotherapy might further increase the strength and rapidity of improvement. Compiling evidence suggests psychotherapy in combination with medication or by itself to facilitate more sustained treatment responses than monotherapy with an antidepressant (Furukawa et al., 2021; Markowitz et al., 2022). The relative inefficacy of antidepressant monotherapy might be partly explained by estimations that more than 90% of SSRIs are prescribed by general practitioners with limited tools or time to empower patients to engage in self-help activities (Ormel et al., 2022). For optimization of benefit, treatment could also target the lifestyle factors mentioned in this review by educating and motivating patients about their power to change their health. For example, engagement in regular exercise can increase BDNF-release, hippocampal volume, and resilience to neuropathology and cognitive impairment (Arida and Teixeira-Machado, 2021), and replacing some of an ultra-processed diet with less processed foods is estimated to reverse the risk of neurodegeneration associated with its intake (Li et al., 2022). It has been argued that mono-treatment without behavioral management might even be counterproductive when it reduces self-help activity and active coping (Meadows et al., 2019). Meadows and colleagues point out that when presented with biomedical, rather than biopsychosocial treatment, depressed patients may transfer agency for recovery, which can reduce engagement in self-help strategies. Ormel et al., 2022 propose that medication should not be administered alone but should be combined with interventions to guide plasticity within the brain, by providing appropriate environmental input such as behavioral activation and meditation techniques. Likewise, an expert review argues that ketamine should only be administered in settings with multidisciplinary personnel (McIntyre et al., 2021).

Like its antidepressant predecessors, ketamine is not likely to be a miracle cure that will guarantee remission without the involvement of active effort and psychosocial care. Although a key strength is its rapid and potent temporary lift of suicidality (Wilkinson et al., 2018), depressive pathology likely demands additional targeting to offer sustainable care. The deeply ingrained pathology involved in depression, especially that of a chronic, recurrent, or treatment-resistant nature, likely needs to be targeted via some of the same factors which induced it. This might include social-environmental experiences facilitating psychological reframing, and lifestyle changes which lower the risk and impact of stress and inflammation.

#### 4.2.3. Leveraging biologically mediated subjective experience

If the subjective experience of the world can influence neuroimmune systems to increase proinflammatory activity in response to perceived threat, then it is not so inconceivable to hypothesize modulation towards safety by the very same systems. As seen in cognitive behavioral therapy, the client’s cognitive-affective schemas which are contributing to depressive dysfunction can be altered by restructuring beliefs – *if* the client is receptive to change. The neuroprogressive factors discussed herein can contribute to a maladaptive spiral working in concert with cognitive-affective schemas, which over time could make the client less receptive to change. If the biological “shackles” maintaining this are broken, and intracellular cascades open up for new neural paths to be made, this opens up a potential of restructuring maladaptive beliefs of the self, world, and future which might have facilitated treatment resistance. Furthermore, ketamine in conjunction with psychotherapy might have stronger effects than monotherapy with conventional antidepressants because of the salient experience it produces, and the therapist’s awareness and utilization of increased plasticity in the days following infusion. It might also facilitate post-traumatic growth (i.e., positive change following highly challenging life crises; Tedeschi and Calhoun, 2004) rather than neuroprogressive disturbances; not only because of the subjective experience it provides (Stewart, 2018) combined with integration and reflection sessions employed in clinics, but also because of its mentioned effects on stress, inflammation, toxicity and degeneration, which might prevent further dysfunction facilitating depression.

### 4.3. Considerations for future research

Investigations into the treatment-prevalence paradox (i.e., the lack of prevalence reduction despite growing accessibility and modes of treatment) suggests that the strength and duration of efficacy are overestimated in clinical studies, does not translate to real-world clinical settings, and are markedly different based on recurrence status (Ormel et al., 2022). This highlights the need to further develop research methods to increase the accuracy of predictions, and serves as a reminder that the studies of ketamine’s efficacy might not translate well into clinical settings. Most of the included ketamine studies in this review are of treatment-resistant individuals receiving doses of 0.5 mg/kg, which strengthens generalizability as this most commonly seen in clinics today (Swainson et al., 2021). On the contrary, the lack of assessments of multiple doses over longer time courses, makes generalizability to clinical practice challenging. Accuracy and generalizability of results might be strengthened by employing more naturalistic and longitudinal designs, investigating patients who are undergoing treatment as usual, and using pre-, post-, and long-term follow-up measures.

Future studies might also benefit from measuring possible mediators and predictors of ketamine’s effects, and of time to relapse, such as inflammatory markers, lifestyle factors, concomitant medication, BMI, BDNF, cortisol, kynurenine pathway metabolites, sex, and whether patients received integration therapy following infusions. In this process, it might be helpful to pay attention to whether factors such as sex and BMI cause significant variation in the other possible mediators of effect. The literature may also benefit from mixed methods designs, which allow for a unique unification of quantitative and qualitative data; To see individual history characteristics in relation to a greater set of data points could add great value into the understanding of the different etiologies and manifestations of depression. This could further serve as valuable tools in targeting and treating individuals who have experienced treatment failure.

## 5. Conclusion

The synthesized literature demonstrates the pathophysiology of depression and TRD, and ketamine’s antidepressant actions to be complex, and not yet fully explored. It can nevertheless be concluded that ketamine’s ability to alter neuroprogressive aspects of depression is a promising finding for the depressed and treatment resistant population – although there is a need for more longitudinal studies and certainty about ketamine’s long-term effects. Stress, inflammation, neurotoxicity, and degeneration of neurons and astrocytes are all factors that can contribute to neuroprogressive worsening in depression, and intriguingly, ketamine demonstrates multidirectional reductions of this pathology. The simultaneous targeting of these factors and rapid onset of antidepressant effects constitutes a novel and multidimensional way of reducing depressive pathology. Importantly, this might offer invaluable and long-awaited relief for treatment-resistant patients, especially if utilized in a clinical setting which optimizes ketamine’s effects by employing tailored therapy (e.g., preparation and integration sessions) and knowledge about ketamine’s discussed mechanisms (e.g., increased stress-resilience and neuroplasticity). Ketamine’s rapid effects on BDNF and synaptogenesis is one of the neurobiological mechanisms which most clearly differentiates it from traditional antidepressants; While these most commonly prescribed modes of treatment are expected to require weeks of administration before BDNF upregulation and reduction of symptoms, ketamine’s acute BDNF upregulation appears to allow for instant antidepressant effect. This makes ketamine a unique and potentially critical tool for clinicians helping acutely suicidal and treatment resistant patients.

## Author contributions

AL and EH co-wrote the manuscript. EH wrote the stress and inflammation section. AL wrote the neurotoxicity and neurodegeneration section. ER and GD supervised this project, and provided multiple revisions, feedback, and recommendations. All authors contributed to the article and approved the submitted version.

## Funding

This manuscript was funded by the University of Bergen, Bergen, Norway; European Research Council AdG #693124 and Helse-Vest #912045.

## Conflict of interest

The authors declare that the research was conducted in the absence of any commercial or financial relationships that could be construed as a potential conflict of interest.

## Publisher’s note

All claims expressed in this article are solely those of the authors and do not necessarily represent those of their affiliated organizations, or those of the publisher, the editors and the reviewers. Any product that may be evaluated in this article, or claim that may be made by its manufacturer, is not guaranteed or endorsed by the publisher.
